# Alveolar bone repair with strontium- containing nanostructured carbonated hydroxyapatite

**DOI:** 10.1590/1678-7757-2017-0084

**Published:** 2018-01-16

**Authors:** André Boziki Xavier do Carmo, Suelen Cristina Sartoretto, Adriana Terezinha Neves Novellino Alves, José Mauro Granjeiro, Fúlvio Borges Miguel, Jose Calasans-Maia, Monica Diuana Calasans-Maia

**Affiliations:** 1 Universidade Federal Fluminense, Faculdade de Odontologia, Laboratório Associado de Pesquisa Clínica em Odontologia, Niterói, RJ, Brasil; 2 Instituto Nacional de Metrologia, Qualidade e Tecnologia, Programa de Bioengenharia, Duque de Caxias, RJ, Brasil; 3 Universidade Federal do Recôncavo da Bahia, Centro de Ciências da Saúde, Santo Antônio de Jesus, BA, Brasil

**Keywords:** Hydroxyapatite, Bone repair, Rats, Strontium, Histomorphometric evaluation

## Abstract

**Objective:**

This study aimed to evaluate bone repair in rat dental sockets after implanting nanostructured carbonated hydroxyapatite/sodium alginate (CHA) and nanostructured carbonated hydroxyapatite/sodium alginate containing 5% strontium microspheres (SrCHA) as bone substitute materials.

**Methods:**

Twenty male Wistar rats were randomly divided into two experimental groups: CHA and SrCHA (n=5/period/group). After one and 6 weeks of extraction of the right maxillary central incisor and biomaterial implantation, 5 μm bone blocks were obtained for histomorphometric evaluation. The parameters evaluated were remaining biomaterial, loose connective tissue and newly formed bone in a standard area. Statistical analysis was performed by Mann-Withney and and Wilcoxon tests at 95% level of significance.

**Results:**

The histomorphometric results showed that the microspheres showed similar fragmentation and bio-absorbation (p>0.05). We observed the formation of new bones in both groups during the same experimental periods; however, the new bone formation differed significantly between the weeks 1 and 6 (p=0.0039) in both groups.

**Conclusion:**

The CHA and SrCHA biomaterials were biocompatible, osteoconductive and bioabsorbable, indicating their great potential for clinical use as bone substitutes.

## Introduction

Implant-supported restoration has been increasingly performed by dentists for both aesthetic and functional reasons. However, when infections, pathological processes, extractions, or congenital and traumatic injuries on the maxilla and the mandible lead to bone loss, dental implant installation might not be the best opition^20^. Therefore, to minimize the loss of alveolar bone, or even restore it, different types of alloplastic grafts have been used, and new biomaterials have been the focus of research aiming to develop bone substitutes.

Among these grafts, HA Ca_10_(PO_4_)_6_(OH)_2_ has been widely used as a bone substitute for approximately 80 years^18^. This ceramic is biocompatible, osteoconductive^15^, similar to the bone and tooth tissue inorganic portions^1^, bioactive, and allows substitutions in its molecular formula and periodic monitoring via imaging because of its radiopacity. Additionally, it is mechanically tough and bioactive, and it is not antigenic, carcinogenic, or toxic.

However, the clinically used HA is not biodegradable and remains at the implantation site for long periods^23^, which limits bone regeneration. The lack of degradation is probably due to the high temperatures during ceramics production^16^ and treatment after synthesis (sintering), which increases the crystallinity and hinders biosorption. With this in mind, nanostructured materials composed of particles smaller than 100 nm with low crystallinity show to be potential alternatives to grafts when produced with non-sintered materials at low temperatures^17^, considering they can imitate biological apatite^12^.

Researchers have chemically modified HA by substituting phosphate groups (PO_4_) or hydroxyl groups (OH) with carbonate (CO_3_) to develop a nanostructured carbonated hydroxyapatite at low temperatures^9^. Under these conditions, the produced biomaterial is similar to stoichiometric HA, but with lower crystallinity and higher solubility, which favors rapid bioabsorption and bone regeneration^17^.

Furthermore, the stability and flexibility of the HA structure enables different ionic substitutions^3^. It is possible to induce the exchange of many cations and anions by modifying the structure of stoichiometric HA to resemble biological apatite^1^. In these techniques, calcium frequently substitutes strontium (Sr^2+^)^10^; despite still being present in lower amounts compared to strontium, calcium alters the crystal structure and some HA properties, including phase stability, solubility, and reactivity^4^, thus decreasing ceramic mechanical strength. Additionally, strontium reduces bone resorption and increases bone formation. The high solubility of HA combined with Sr^2+^ increases the number of interconnected pores, promoting cell migration, interfacial bonding^13^, and osseointegration^11^ ^,^ ^18^.

Thus, this study evaluated histomorphometric bone repair in rat tooth sockets after implanting CHA and SrCHA synthesized at low temperatures.

## Material and methods

Animal experiments and breeding were performed according to the institutional review board (CEUA/ UFF), N°179/2012, the NIH Guide for Care and Use of Laboratory Animals and the Brazilian legislation on animal research.

### Biomaterials

The nanostructured carbonated hydroxyapatite powder and nanostructured carbonated hydroxyapatite containing strontium were prepared using a precipitation wet method with average temperature of 5°C and 6% wt CO_3_. The synthesized solids were filtered and washed with deionized water (MilliQ^®^, Millipore Corporation, Billerica, MA, USA) until reaching neutral pH (pH = 7). Then, the material was lyophilized in a FreeZone 1 lyophilizer (Labconco^®^, Kansas City, Missouri, USA) and separated on sieves according to particle sizes from 74 μm to 37 μm.

To produce microspheres, the obtained powders were individually mixed with a solution of sodium alginate (Sigma Aldric^®^/Fluka Biochemika^®^, Buchs, Switzerland) diluted in ultrapure water (MilliQ^®^, Millipore Corporation, Billerica, MA, USA) at a ratio of 15:1. The mixture was then extruded into 0.15 molar calcium chloride (0.15 M CaCl_2_), in which instant microsphere formation was observed. The mixture was kept at rest for 24 h until complete gelation. After this step, the microspheres were washed with ultrapure water until fully eliminating saline. Immediately after the washing, the microspheres were dried by lyophilization for 24 h and separated on a stainless steel sieve with a particle size from 425 μm to 600 μm. The microspheres were then separated into aliquots in an Eppendorf tube and sterilized with gamma radiation in a cobalt-60 irradiator (Gamma Cell) for 760 m with total dose of 15 kGy and dose rate of 19.72 Gy/m. Electron microscopy scanning of the microspheres showed similar morphology and surface texture. SrCHA presented fewer surface pores than CHA, as previously shown^25^.

**Figure 1:**
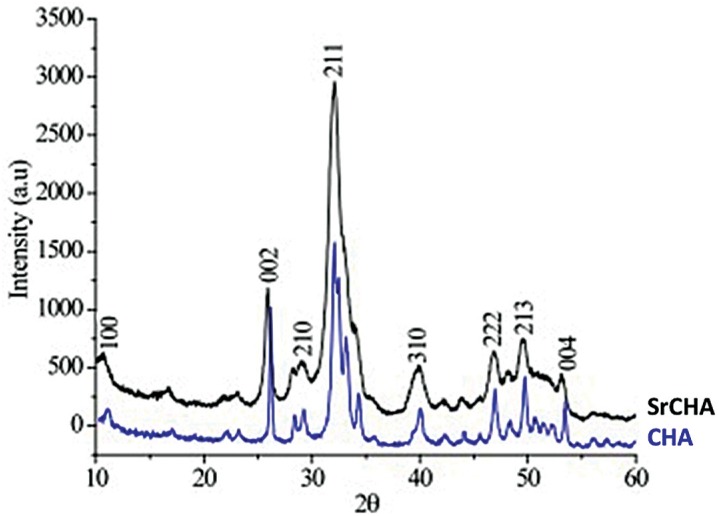
XRD pattern of CHA and SrCHA. The peaks of SrCHA are narrower than those of CHA, indicating low cristallinity for SrCHA when compared to the CHA group

The XRD patterns revealed that the microspheres had low crystallinity, as indicated by the broad and poorly defined peaks. However, the XRD pattern of SrCHA (Figure 1) showed narrower peaks than that of CHA because of the presence of Sr.

**Figure 2:**
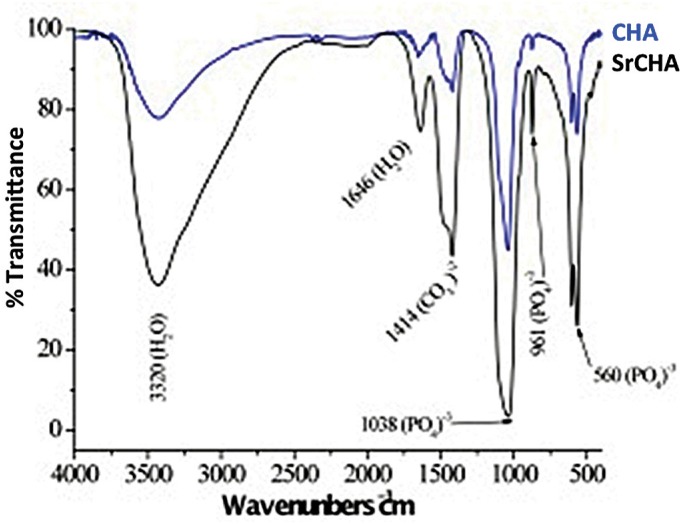
Infrared Fourier transform spectrum of CHA and SrCHA. We observed major bands regarding phosphate and carbonate groups, a characteristic of carbonated hydroxyapatite. Presence of water bands, indicating the material is not ceramic (regions 867, 868, 1415, 1425, and 1482 cm^−1^)

The spectra in Figure 2 show the vibrational bands correspond to CHA. The 3435 and 1639 cm^−1^ regions, which are large and intense, represent water bands, indicating that the material is not ceramic. In Figure 2, regions 867, 868, 1415, 1425 and 1482 cm^−1^ show carbonate ions, indicating that replacement occurred as expected. The other bands that can be observed in the figure show phosphate ions. Because of the high hydration of the samples, it was not possible to identify hydroxyl ions representative bands.

### Animals and surgical procedure

A total of 20 male Wistar rats with average weight of 300 g were randomly divided into two groups (CHA and SrCHA) with five animals for each experimental period. The sample size was based previous studies that followed the same animal protocol^16^. According to the CONCEA and 3R's program, we should reduce the number of animals in experimentation as much as possible without loosing the accuracy of the statistical analysis. Five animals for each group is the minimum to perform the normality test^21^. The animals were evaluated after one and six weeks.

**Figure 3:**
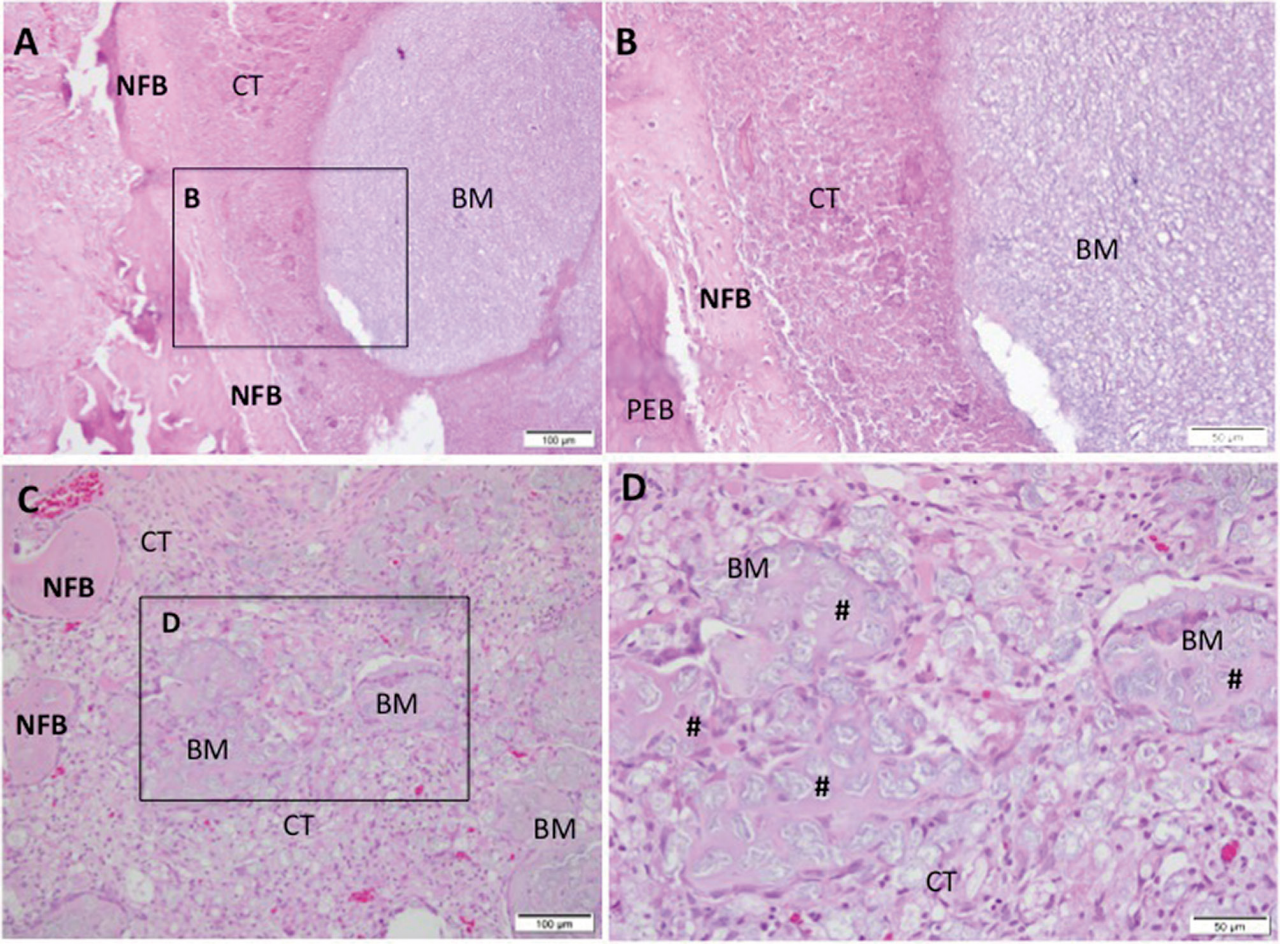
Representative photomicrographs of alveolar socket after 7 days. A and B: CHA group and C and D: SrCHA group. In A and B: presence of connective tissue surrounding the biomaterial (BM) microsphere and peripheric newly formed bone (NFB). C and D: presence of newly formed bone surrounding the connective tissue area, containing particulate biomaterial with peripheric osteoid (#). Square with 40-fold magnification taken from A and C, respectively. NFB: Newly Formed Bone; BM: Biomaterial; CT: Connective Tissue; #: Osteoid. Hematoxylin and eosin stained. Bar: A and C: 100 μm; B and D: 50 μm

After anesthesia with intramuscular injection of 75 mg/kg ketamine hydrochloride (Ketalar^®^, Veltbrands, São Paulo, Brazil) and sedation with 1.5 ml/kg xylazine (Rompun^®^, Veltbrands, São Paulo, Brazil), the antisepsis of the perioral region and oral mucosa was achieved with 2.0% and 0.12% chlorhexidine, respectively. Then, sindesmotomy and extraction of the upper right central incisor were performed with a dental explorer no. 5 (Duflex^®^, São Paulo, SP, Brazil) and a pediatric forceps no. 151 (Duflex^®^, São Paulo, SP, Brazil), respectively (Figure 3A and 3B), and 0.2 mg of the biomaterials (Figure 3C) was implanted in the tooth socket, followed by suturing (Figure 3D). After the surgical procedures were completed, postoperative analgesia with 1 mg/kg meloxicam (Duprat^®^, Rio de Janeiro, Brazil) was administered subcutaneously every 24 h for three d since the day of the surgery.

After the experimental period, the animals were euthanized by applying a lethal dose of thiopental 150 mg/kg [(Thiopentax^®^ (Cristália), Itapira, São Paulo, Brazil)] to collect bone blocks containing the biomaterials and surrounding tissues. The specimens were fixed in 4% formaldehyde, decalcified in decalcification solution (Allkimia^®^, Campinas, São Paulo, Brazil) for 48 h and embedded in paraffin. The blocks were cut at 5-μm thickness, stained with hematoxylin and eosin (HE) and examined by light microscopy (Eclipse E400, Nikon^^®^^, Tokyo, Japan).

### Histomorphometric analysis

Histomorphometric analysis was performed to quantify the remaining biomaterial, the connective tissue loose and the bone newly formed in the standard area. The morphometric measurements were performed using the Image-Pro Plus^^®^^ software, version 4.5.0.29 (Media Cybernetics, Silver Spring, EUA) with 5 microscopic fields under 20x augmentation with a 1-blinded examiner (SCS). In each histological slice stained with HE, 5 non-superimposing microscopic fields obtained by scanning at 20x magnification were captured in the medium third region of the socket after biomaterial implantation. With the Image-Pro Plus^®^ 6.0 (Media Cybernetics, Silver Spring, Maryland, USA), a grid of 250 points superimposed on the area under analysis allowed the determination of the volume density of the newly formed bone, of the connective tissue and of the residual biomaterial. The 250 points superimposed on each photomicrograph were considered as 100%, so each point was classified and the percentage of each parameter was obtained.

### Statistical analysis

The results are presented as percentages, and values are presented as the mean value (±) standard deviation. The mean values and standard deviations obtained in each group were tested for normality according to D'Agostino-Pearson's omnibus test. The data did not present a normal distribution, this way both the non-parametric statistical analysis of Mann- Withney for inter-group and and the Wilcoxon for intragroup were performed using the Prism Graph Pad 6.3 software (Inc. La Jolla, California, USA), with p≤0.05 being considered statistically significant.

## Results

### Histological results

#### 1 week

At the first experiment, both biomaterial groups presented the dental socket filled with fragmented biomaterial spheres surrounded by granulation tissue with remnants of blood clots, neoformed capillaries, and fibroblasts. In both groups, we observed moderate mononuclear inflammatory infiltrate as lymphocytes and macrophages between the components of the conjunctive tissue and the surroundings of partially bioabsorbed microspheres. We also observed giant cells permeating the biomaterial particles. New bone formation occurred centripetally in patches during this period and was more evident in the apical region of the socket (Figure 3).

#### 6 weeks

**Figure 4:**
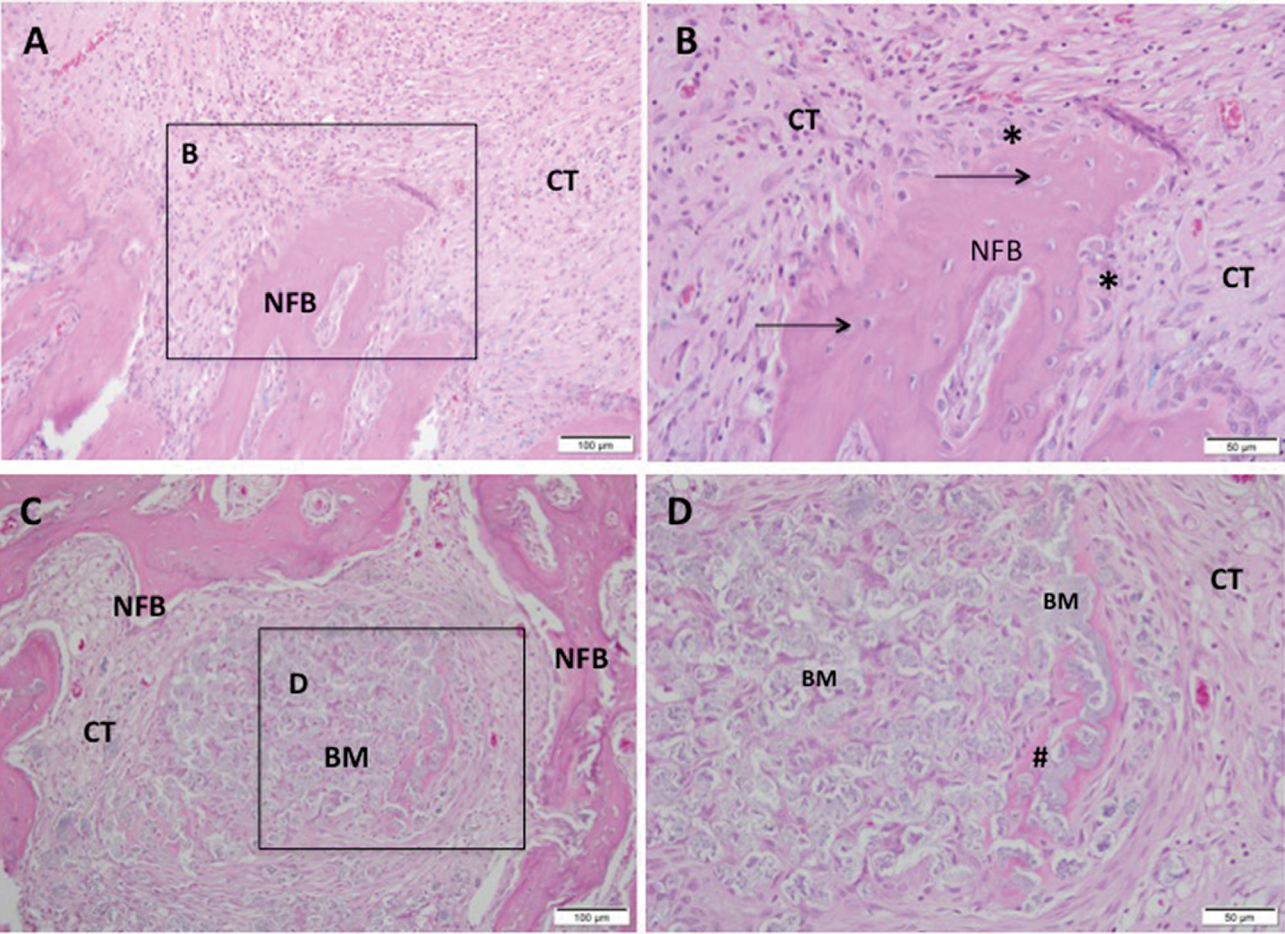
Representative photomicrographs of alveolar socket after 42 days. A and B: CHA group and C and D: SrCHA group. A and B: observe the presence of newly formed bone (NFB) with osteocytes (arrow) and osteoblastic paving (*) permeated by fibrocellular connective tissue (CT), with few inflammatory cells and absence of biomaterial (BM). C and D: presence of new-formed bone with connective tissue at the surroundings containing fragmented biomaterial and peripheric osteoid (#). A and C: 20-fold magnification and B and D: square with 40-fold magnification taken from A and C, respectively. BM: Biomaterial, NFB: Newly Formed Bone, CT: Connective Tissue; #: Osteoid, *: Osteoblasts. Hematoxylin and eosin stained. Bar: A and C: 100 μm; B and D: 50 μm

In both groups, we observed a discrete chronic inflammatory response, with a few giant cells close to the microspheres, which were evidently fragmented. The biomaterials were fragmented and bioabsorbed differently. The quantity of remaining material in the SrCHA group was slightly lower than that from the CHA group. Bone formation process replaced the inflammatory cell content in the first experiment at the same time that there was a decrease in mature connective tissue composed by collagen fibers. The newly formed bone characterized by the thick trabecular bone was similar in both groups and occurred near the remaining bone and in direct contact with microspheres (Figure 4).

The histological evaluation showed that in the interstitial spaces formed between the microspheres with no bone formation had a highly vascularized loose of connective tissue after one week that showed increased organization after six weeks with newly formed bone. This angiogenesis is essential for bone regeneration because these new blood vessels provide oxygen, nutrients, and cells, all considered essential for bone formation.

### Histomorphometric evaluation

**Figure 5:**
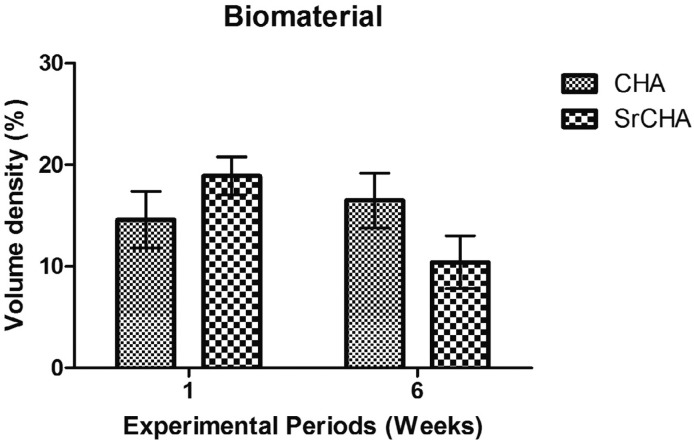
Volume density of the remaining biomaterial in the dental alveolus after 1 and 6 weeks of implantation. The values were similar for both groups in both experimental periods. We observed no significant difference between groups. Results are shown as mean percentages ± confidence intervals (vertical bars)

**Figure 6:**
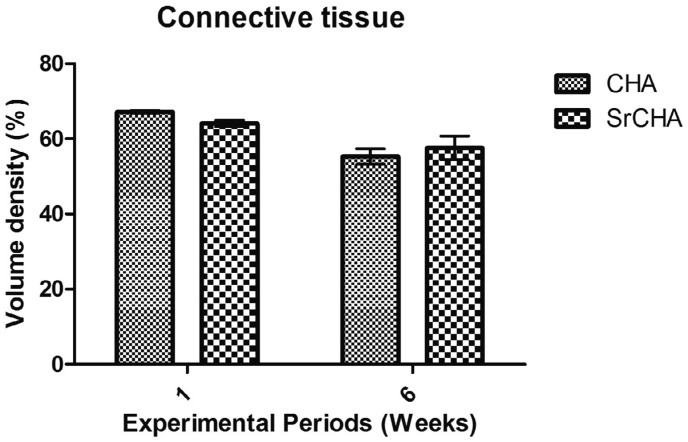
Volume density of connective tissue in the tooth socket after 1 and 6 weeks of implantation. The values were similar for both groups in both experimental periods. We observed no significant difference between groups. Results are shown as mean percentages ± confidence interval (vertical bars)

**Figure 7:**
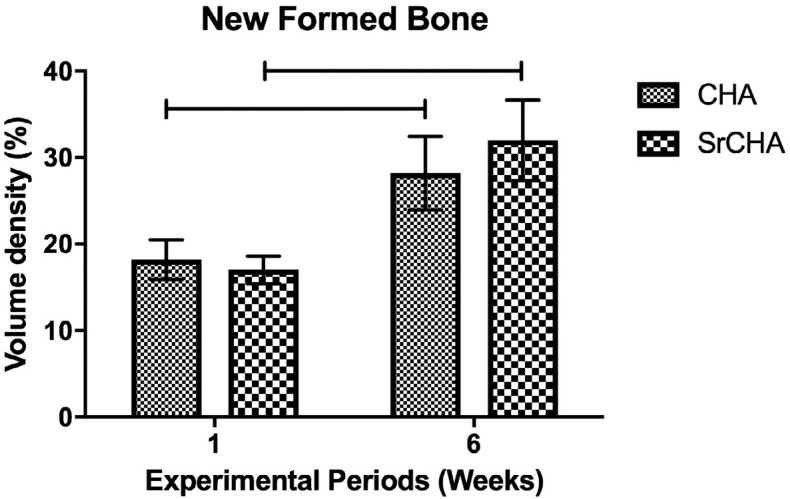
Volume density of newly formed bone. The quantity of newly formed mineralized tissue was similar in both groups in both experimental periods. However, we observed more newly formed bone at 6 weeks than at 1 week. The horizontal lines between bars indicate significant differences between groups (p<0.05). Results are shown as mean percentages ± confidence interval (vertical bars)

Histomorphometric analysis showed no significant differences after 1 (CHA=14.6±2.50 and SrCHA=18.9±1.69) and 6 weeks (CHA=16.5±2.41 and SrCHA=10.4±2.33) between groups regarding biomaterials bioabsorption (Figure 5). The amount of connective tissue formed in the tooth socket permeating the microspheres was similar for both groups and periods (Figure 6). Regarding the newly formed bone, morphometric analysis showed that the percentage of mineralized tissue was similar between groups at 1 (CHA=18.2±2.04 and SrCHA=17±17, and 6 weeks (CHA=28.2±3.82 and SrCHA=32±4.15). However, we observed a higher percentage of newly formed bone in both groups after 6 weeks when compared to1 week (p=0.0039) (Figure 7).

## Discussion

Calcium ions in biological apatite have been partially substituted with other ions, such as Sr^2+^, Mg^2+,^ and Zn^2+^. This change affects the crystallinity, solubility, surface energy, and dissolution rate of the material, thus improving its bioactivity. Based on these findings, this study evaluated a promising bone substitute based on a synthesized at low temperature nanostructured carbonated hydroxyapatite containing strontium^10^.

According to a previous study^5^, the sintering of nanostructured hydroxyapatite causes crystal densification and nanostructured features loss, thereby increasing crystallinity and reducing solubility^15^. Additionally, the sintering process is responsible for the removal of sodium alginate. In this study, we did not sintered the materials, retaining their nanometric characteristics, low crystallinity and sodium alginate content.

The non-strontium-containing hydroxyapatite was used as control group, as we aimed to evaluate the influence of strontium on hydroxyapatite in bone repair. We did not perform the dental socket filling with blood clot, as these results have already been published with the same experimental periods^22^ and are already established in the literature^19^. In addition, according to the recommendation by the CONCEA and the 3R's Program^21^, the number of animals used in experimental studies should be reduced.

The biomaterials evaluated in this study were biocompatible, despite the presence of a limited chronic inflammatory response with giant cells that diminished with time. The giant cells, considered as foreign body type giant cells, are of monocyticphagocytic lineage and are important in the tissue repair mechanism, as they carry out cellular and tissue debris phagocytose, as well as contribute to the bioabsorption of biomaterial fragments, besides secreting cytokines that favor essential cellular events in tissue repair. The presence of multinucleated giant cells modulated by the chemical surface of the biomaterials^2^ has demonstrated the importance of macrophage subsets in the reaction to foreign bodies and, consequently, in the biocompatibility of biomaterials^6^ ^,^ ^7^. Therefore, the presence of these cells can occur as an attempt to reabsorb the material, which not necessarily implies lack of biocompatibility. From a biological point of view, the material fragmentation caused by lower crystallinity and smaller particles susceptible to phagocytosis could justify the presence of these cells around the particles. This type of tissue response is considered inherent to the healing mechanism after the implantation of biomaterials^14^ and has been observed in other studies on bone regeneration with biomaterials^14^.

A previous study observed that at the nanoscale, the biomaterial resembled biological apatite^5^ and presented bioabsorption similar to that observed in other studies^15^ ^,^ ^19^. Such bioabsorption is an essential for a suitable bone substitute because biosorption is important for bone physiology after biomaterial implantation^15^. In areas where the microspheres were bioabsorbed, we observed the formation of mineralized tissue, characterized by more cellular and non-lamellar bone associated with microspheres and at contact with the remaining alveolar bone, delimiting the dental alveolus, which confirms that the nanostructured carbonated HA here evaluated was bioactive, osteoconductive^16^ ^,^ ^19^ and highly crystalline^5^ ^,^ ^23^.

The use of biomaterial microspheres has been considered preferable because the interstitial space between the implanted spheres provides macropores for tissue invasion and also because microsphere implantation can be performed with minimally invasive surgical techniques. In addition, the spheres do not have surface edges or dimensions that could lead to inflammation. To produce the microspheres, we mixed nanostructured carbonated HA powders with sodium alginate, an inert and biodegradable polymer. The histological results showed that there were interstitial spaces between the microspheres. In areas where there was no new bone formation, we observed a highly vascularized and loose connective tissue after one week that showed increased organization after six weeks. This angiogenesis is essential for bone regeneration because these new blood vessels provide oxygen, nutrients, and cells considered essential for bone formation^24^.

In the histological evaluation, we observed a greater fragmentation of SrCHA compared to the CHA group. We did not sinter the biomaterials used in this study, so they are considered low crystalline materials. However, the incorporation of strontium in the hydroxyapatite by partially replacing it with calcium changed crystallinity, morphology, lattice parameters, crystal size, stability, bioactivity, biocompatibility, and osteoconductivity of CHA. This set of physical and chemical changes alter the fragmentation and bioabsorption of biomaterials^25^.

Sr^2+^ has been widely used for partial substitutions of HA because of its dual ability to stimulate bone formation and reduce bone resorption. However, in our study, we observed no increase in newly formed mineralized tissue in the SrCHA group, indicating that Sr^2+^ did not influence the osteogenic potential of nanostructured carbonated HA, possibly because of the low Sr^2+^ content (1.7%) identified by atomic absorption spectrometry after synthesis and prior to the implantation, regardless of an initial theoretical Sr^2+^ concentration of 5%. These results are similar to those obtained by other study^8^ aimed at producing 1% ZnHA, obtaining a maximum incorporation of 0.4%. Similarly, Resende, et al.^23^ (2013) showed a reduction of approximately 50% in the experimental zinc concentration of ZnHA compared to the initial theoretical concentration.

## Conclusion

Our results suggest that both CHA and SrCHA, produced at low temperature and not sintered, were biocompatible, bioactive, ostecondutive osteoconductive, and bioabsorbable, indicating its great potential for clinical use as bone substitutes. Further studies with a higher content of Sr^2+^ associated with nanostructured carbonated hydroxyapatite are necessary to evaluate the effect of Sr^2+^ on the biological response.
